# The Functional Human C-Terminome

**DOI:** 10.1371/journal.pone.0152731

**Published:** 2016-04-06

**Authors:** Surbhi Sharma, Oniel Toledo, Michael Hedden, Kenneth F. Lyon, Steven B. Brooks, Roxanne P. David, Justin Limtong, Jacklyn M. Newsome, Nemanja Novakovic, Sanguthevar Rajasekaran, Vishal Thapar, Sean R. Williams, Martin R. Schiller

**Affiliations:** 1 Nevada Institute of Personalized Medicine, and School of Life Sciences, University of Nevada, Las Vegas, Nevada, United States of America; 2 Department of Computer Science and Engineering, University of Connecticut, Storrs, Connecticut 06269–2155, United States of America; 3 Department of Pathology, Massachusetts General Hospital, Boston, Massachusetts 02114, United States of America; University of Rome Tor Vergata, ITALY

## Abstract

All translated proteins end with a carboxylic acid commonly called the C-terminus. Many short functional sequences (minimotifs) are located on or immediately proximal to the C-terminus. However, information about the function of protein C-termini has not been consolidated into a single source. Here, we built a new “C-terminome” database and web system focused on human proteins. Approximately 3,600 C-termini in the human proteome have a minimotif with an established molecular function. To help evaluate the function of the remaining C-termini in the human proteome, we inferred minimotifs identified by experimentation in rodent cells, predicted minimotifs based upon consensus sequence matches, and predicted novel highly repetitive sequences in C-termini. Predictions can be ranked by enrichment scores or Gene Evolutionary Rate Profiling (GERP) scores, a measurement of evolutionary constraint. By searching for new anchored sequences on the last 10 amino acids of proteins in the human proteome with lengths between 3–10 residues and up to 5 degenerate positions in the consensus sequences, we have identified new consensus sequences that predict instances in the majority of human genes. All of this information is consolidated into a database that can be accessed through a C-terminome web system with search and browse functions for minimotifs and human proteins. A known consensus sequence-based predicted function is assigned to nearly half the proteins in the human proteome. Weblink: http://cterminome.bio-toolkit.com.

## Introduction

Minimotifs, also called short linear motifs (SLiMs), are contiguous 2–15 amino acid sequences with a known molecular or cellular function in at least one protein [1]. The functions of minimotifs include post-translational modifications (PTMs), binding to other proteins and molecules, and protein trafficking. Our laboratory has built one of the initial minimotif search systems, Minimotif Miner [2–4]. Through working with minimotifs for a decade we have recognized a pattern wherein the carboxylic acid terminus (C-terminus) of many different proteins contains one or more minimotifs. We designate the C-terminus as the last 10 amino acids of a protein chain. Although minimotifs can be present anywhere in the sequence, some are functionally important only when present at the C-termini of the protein. For example, the KDEL> sequence (single letter amino acid code; > indicates the C-terminal end) is a trafficking signal found on soluble endoplasmic reticulum-resident proteins and was one of the first minimotifs identified [5]. There seems to be a strict requirement for C-terminal minimotifs being at the C-terminus because it is difficult to identify C-terminal minimotif sequences that are functional when located elsewhere in the protein. For example, the SKL> peroxisomal targeting minimotif does not target when not located at the C-terminus [6].

In considering the role of the C-terminus in proteins, there are four main areas of research identifying C-termini: (1) sequences, herein called instances; (2) consensus sequences that are found in many proteins; (3) functions, also called activities; and (4) structures. C-termini are generally formed from nascent transcription and translation producing proteins with a carboxylic acid end. New C-termini can be introduced by proteolysis revealing masked C-termini, and alternative splicing can produce protein isoforms with different C-termini derived from the same gene. Alternative splicing often alters minimotifs in proteins [7]. Other molecular changes of C-termini can result by enzymatic addition of PTMs or by post-transcriptional RNA editing resulting in an amino acid substitution. We consider the C-terminal sequence and minimotif functions to be the “C-terminome”.

Most C-termini are identified by *in silico* transcription/translation of genomic DNA sequences. Many C-termini are captured in the predicted proteomes of major protein sequence databases, such as RefSeq and UniProt [8,9]. Using this data, C-terminal sequences in yeast and other organisms have been identified [10]. TopFIND has also enumerated the C-termini generated by proteolysis for several proteomes [11]. New C-termini arising from alternative splicing are included in databases such as TopFIND, RefSeq, AST, ASTD, and H-DBAS [12–15]. While based upon predicted proteomes, mass spectrometry-based approaches can be used for direct assessment of C-termini in a proteome, such as that reported for *E*. *coli* [16].

Much effort has focused upon discovering novel sequence patterns found at high frequency on the C-termini of multiple proteins. This is thought to imply function, a concept used by COPS, MOTIFS and PRINTS for signature patterns and is supported, at least in some cases, by the discovery of previous known functional C-terminal minimotifs [17–22]. Several groups have searched for novel peptide patterns on the C-terminus ranging from 3–10 residues, and have identified repetitive patterns such as the DSD sequence [20–23].

One general limitation of these studies is that most minimotif consensus sequences in Minimotif Miner 3 (MnM) and Eukaryotic Linear Motif (ELM) have multiple positions of degeneracy, where most or all amino acids can be functionally tolerated at more than one position in the minimotif [24]. ProTeus uses a variation called gapped-SIGs, which allows one degenerate position in C-terminal sequence up to 10 amino acids in length [21]. While these studies have identified instances in C-termini that match highly recurrent consensus sequences, the amount of degeneracy used does not match that for a typical minimotif consensus sequence. Furthermore, very little is known about the molecular functions of these consensus sequences. An analysis that allows for more degeneracy and provides a means to investigate the functions of identified consensus sequences is needed.

**Fig 1:**
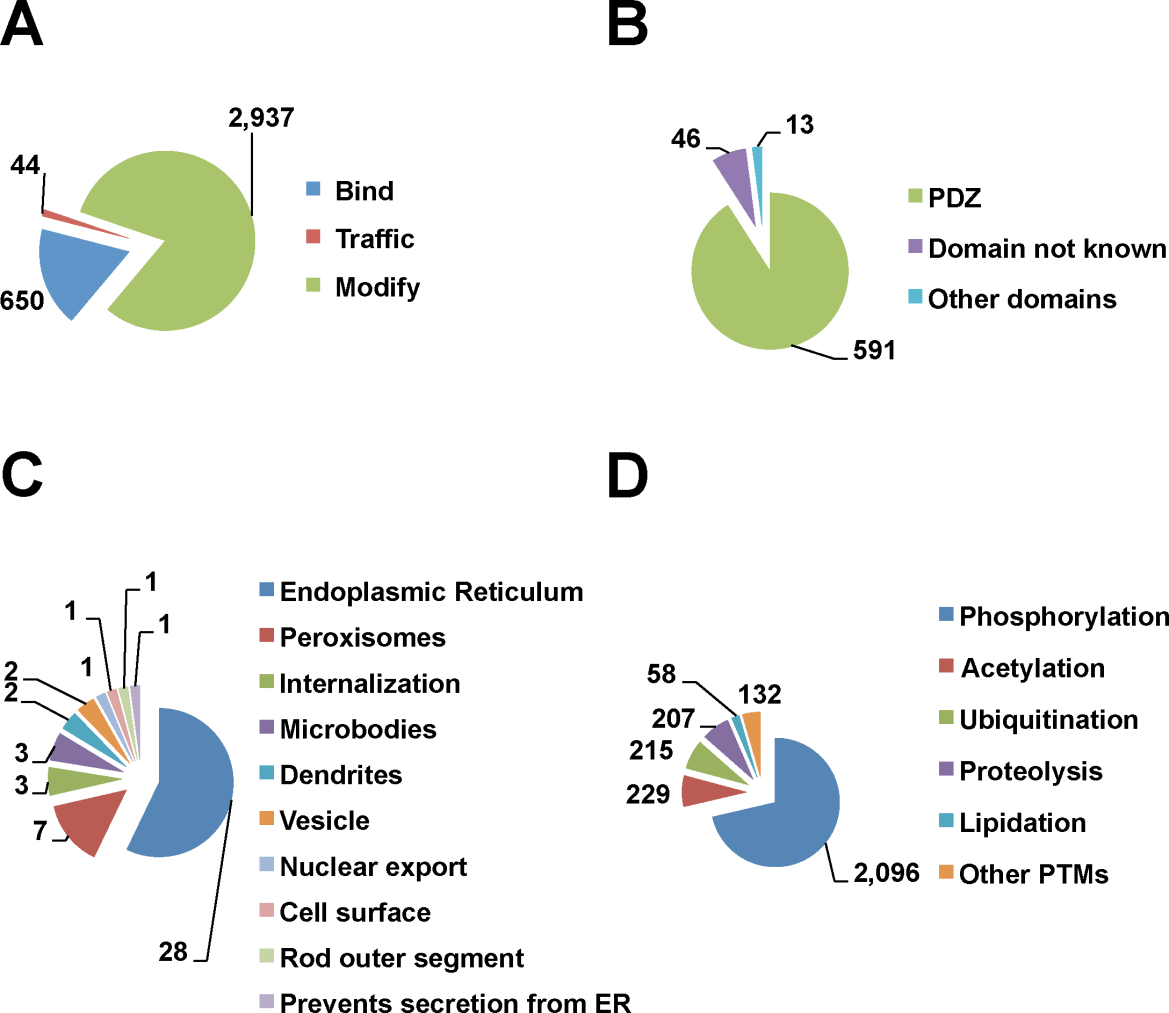
Functional landscape of C-terminome. **(A)** A pie chart shows the number of experimentally verified C-terminal minimotifs with different functions. The molecular function for each category is shown in the other panels: bind (**B**), traffic (**C**), and modify (**D**). This graph includes both consensus sequences and instances. **(B)** A pie chart showing the types of domain targets for binding minimotifs. The “Other domains” category includes Transducin-like enhancer proteins, Tetratricopeptide repeat domain, 14-3-3 domain, G protein coupled receptor, COPI and COPII binding proteins, and ubiquitin- binding proteins. The category 'domain not known' indicates that the specific interaction domain of the target protein was not identified. **(C)** A pie chart showing the different compartments for trafficking motifs. **(D)** A pie chart showing functional categories for post-translational modifications. The “other PTMs” category (1%) includes methylation, prenylation, glycosylation, crotonylation, amidation, farnesylation, sulphation, de-phosphorylation, o-glcnacation, geranyl-geranylation, glycation, carboxy-methylation, deamination, sumoylation, tri-iodination, malonylation, mevalonation, and palmitoylation.

Through inspection of the Minimotif Miner database, we recognized that many proteins in the human proteome have verified C-termini functions. Modular protein domains such as PDZ, TPR, and PTB bind specifically to C-terminal minimotif sequences [25,26]. These domain interactions are often involved in binding and protein trafficking [25,27]. For PDZ binding motifs alone, there are about 100s C-termini instances that have been identified [28]. Several algorithms have been used to predict instances for specific C-terminal patterns for functional minimotifs, such as for binding PDZ domains and Peroxisome Targeting Signals (PTS) [28–30]. However, no single source exists that consolidates the functional information of the C-termini of human proteome. The MnM database and ELM resource have C-terminal minimotifs, but have a broader focus on consensus sequences distributed throughout proteins [2–4,31]. While TopFIND has information on C-terminus of proteins, this database focuses more on proteolytically generated new C-termini, rather than the function of the motifs; it contains only 37 instances for four PTMs [11]. With the increase in the discovery of C-termini consensus sequences associated with functions **(Fig 1)**, a database dedicated to the C-termini of proteins and their functions is needed.

In considering the function, it is also relevant to consider the 3D structures of C-termini. Being on the ends of proteins, the C-terminus is most often solvent-accessible when structures from Protein Data Bank (PDB) are examined [32]. Furthermore, the C-termini do not often show electron density in structures determined by X-ray and do not have Nuclear Overhauser effects (NOEs) in structures determined by Nuclear Magnetic Resonance (NMR) spectroscopy. This suggests that many C-termini are poorly structured; however, many structures of C-terminal minimotifs bound to proteins do show structure. For example, PDZ-binding minimotifs bind to a PDZ domain by β-strand addition, whereby a β sheet of the PDZ domain is extended by the minimotif [33]. Thus, it appears that many C-termini may initially be poorly structured, but assume an induced fit upon binding [24].

Here, we report the human C-terminome database and web system that will help scientists explore the functional role of C-termini in proteins of interest. The database contains thousands of C-terminal minimotifs with known function. Functions of other C-termini can be investigated based upon predictions inferred from experiments in rodents, predicted from known consensus sequences for minimotifs functions, and from new anchored consensus sequences on the C-terminus. The C-terminome web system enables a new approach for connecting proteins with poorly understood functions to other proteins that have more established roles in molecular reactions, pathways, or cell processes.

## Materials and Methods

### Data sources

Several databases were used to build the C-terminome database. The Minimotif Miner 3 (MnM 3) database was used as a starting source for experimentally verified C-termini minimotifs. Additional annotation content for minimotifs was extracted from the PubMed, PhosphoSite Plus, and UniProt databases [9,34,35]. The PDB was used to identify structures of C-termini minimotifs [36]. The RefSeq protein database provided sources of proteins, sequences and alternatively spliced proteins [37]. MnM3 database and research articles in PubMed were sources minimotifs in rodent proteomes [4,35].

### C-terminal minimotif instances and consensus sequences

A minimotif instance is a short contiguous peptide sequence in a protein with a demonstrated experimental function. A consensus sequence is short contiguous peptide sequence that represents the minimal common sequence of multiple functionally related instances and often has at least one position of degeneracy. A predicted C-terminal minimotif is any C-terminal sequence whose function has not yet been experimentally tested.

### Predicting functions of C-terminal sequences based upon minimotif consensus sequences and instances

The C-terminus of the C-terminome database was queried to identify all sequences matching a minimotif consensus sequence. All the matched instances that had experimental evidence for a consensus sequence were then eliminated to generate a list of predicted instances. Predictions based on consensus sequences are only based on the presence of the matched sequences that do not yet have a defined minimotif function.

### Predicting functions of C-terminal sequences based on minimotifs in rodent proteomes

The data on C-terminal minimotifs in mouse and rat proteomes were extracted from the MnM3 database [4]. The 10 C-terminal amino acids in rodent proteins having a C-terminal minimotif were manually aligned to their human ortholog. The existence of each C-terminal rodent minimotif in the human proteome was checked manually by aligning the C-terminal region having the minimotif sequence of the protein homologs.

### Identification of *de novo* C-terminal instances, consensus sequences, and occurrences

A detailed description of the algorithm used to create *de novo* sequences is in supplementary methods. Briefly, for each protein in human proteome, anchored C-terminal sequences from 3–10 amino acids long were used to make combinations of consensus sequences with 0–5 degeneracies while retaining the same first and the last amino acid as in the original sequence. Consensus sequences that did not have more than one representative C-terminal sequence were removed. The resulting combinatorial set was termed "*de novo* sequences". In addition to the *de novo* consensus sequences and instances, occurrences also include matches to these sequences. These categories do not include the minimotifs and the predicted minimotifs based on consensus sequences and rodent proteomes. If a particular instance or consensus sequence does not exist at the C-terminus of any protein in the human proteome, then it does not appear in the C-terminome database or website. 9,283,432 unique predicted instances (including consensus sequences) were identified using the human proteome for both reference and spliced C-termini.

### Calculation of fold enrichment

Proteome-wide and discrete proteome enrichment scores for C-termini minimotifs and *de novo* sequences were calculated by generating 100 random proteomes. The random proteomes were of the same size, the same amino acid composition, and had the same length distribution as the C-terminal region of the reference proteome. The overall amino acid composition of the C-terminal region was found to be similar to the entire reference proteome (**S1 Fig**). The proteome-wide fold enrichment score for each minimotif was calculated by dividing the number of times the minimotif was observed in the human proteome at the C-terminal region by the number of times it was observed at the C-terminal region of random proteomes. To account for the same minimotifs present in the C-terminal region of spliced variants, we calculated the discrete-proteome fold enrichment, the number unique proteins not inclusive of spliced variants with the same C-termini. The discrete-proteome fold enrichment for each minimotif was calculated by dividing the number of times the minimotif was observed in the human proteins with distinct C-terminal region by the number of times it was observed at the C-terminal region of random proteomes. A Mann-Whitney U test was performed to determine if there were any statistically significant difference between the fold enrichment scores calculated from true positive (TP) and the true negative (TN). Both TP and TN were identified from the primary literature. A TP was defined as a minimotif sequence with a demonstrated molecular function through experimentation. A TN is defined as the mutant protein with the eliminated function from the same experiment.

### Genome Evolutionary Rate Profiling scores

Genome Evolutionary Rate Profiling (GERP) scores were obtained from the USCS Genome Browser and are a statistic that measured evolutionary constraint [38–40]. GERP scores for the last 10 amino acids for each protein in the proteome are used on the C-terminome web system.

To evaluate whether GERP scores had any predictive value for minimotifs, we analyzed four minimotifs: SKL> targets proteins to Peroxisomes, KDEL> retains proteins in the Endoplasmic Reticulum, VPV> binds PDZ domains, and C[GAVLI][GAVLI]x> is prenylated. Positive predictive value (PPV) and accuracy (Eqs 1 and 2) were used to assess which GERP score threshold produced the best quality predictions.

$$PPV\;(\%)=\frac{n}{n+m}\times 100$$ (1)

$$Accuracy\;(\%)=\frac{n+p}{n+m+p+q}\times 100$$ (2)

where n is the number of true positives, m is the number of false positives, p is the number of true negatives, and q is the number of false negatives.

For this analysis, the assumed TNs for SKL> or KDEL> minimotifs were based on the observation that proteins containing these minimotifs had a defined subcellular localization in the UniProt database, but no peroxisomal or endoplasmic reticulum sub-cellular localization, respectively (n = 11) [9].

### Identifying variants in C-terminal minimotifs

Allele frequencies of C-terminal minimotifs were obtained from the 1000 genomes project phase I call sets [41]. Mapping of variants to minimotifs and their allele frequencies was as described [39]. Briefly, the mapping of SNPs to the C-terminal region of proteins was accomplished by first assembling a generic proteome defined by the reference genome GRCh37 and the Ensembl database of exons and their positions on the reference genome [42]. The predicted protein sequences were verified by aligning reference genome proteins to RefSeq proteins. Finally, the effects of SNPs on C-terminal minimotif amino acid substitutions was predicted by substituting the variant nucleotide observed in the 1000 genomes project for the corresponding nucleotide defined by the reference genome.

### Software engineering

The C-terminome web-application was built based on a standard three-tiered software architecture. The backend is comprised of a logic layer written in Java, as well as data layer with data stored in MySQL tables. The user-interface presentation layer was coded in JavaScript. For structure and styling of user-interface, HTML and CSS was used. The front-end and back-end were connected through AJAX.

## Results

### C-terminome database

The goal of the C-terminome web application is to consolidate knowledge about the functions of protein C-termini encoded by minimotif sequences, and to predict new functions of C-termini in the human C-terminome. A variety of external databases were used as data sources for our C-terminome database. Statistics for the databases are shown in **Table 1**. The C-termini of all proteins in the human proteome were obtained from RefSeq protein records having 35,581 proteins inclusive of 19,522 alternative spliced variants, another source of substituted C-termini [8]. The spliced variants were inferred by cross-referencing gene IDs of all protein entries.

**Table 1:** Summary statistics of the C-terminome database.

C-terminome statistics	Number
**Protein C-termini**	
Protein C-termini (RefSeq)	16,059
Protein C-termini, alternative splice variants (RefSeq)	19,522
Total C-termini	35,581
**Minimotif Sequences**	
Experimentally verified motif instances	3,593
Predictions—inferred from rodents	867
Predictions—by consensi	27,546
Predictions—*de novo* consensus sequence and instances	9,283,432
Total predicted sequences	9,311,845
**Minimotif activities**	
Binding	650
Modification	2,937
Trafficking	44
Total functions	3,631

To assign functions to the C-termini, several sources were used to identify instances and consensus sequences that describe the known functions of the C-terminus on each gene and its spliced variants. A set of instances is often used to extract consensus sequences, which describe the critical residues necessary for the function. These are generally modular, with instances in multiple proteins. MnM3 contains a curated a set of ~550,000 functional minimotifs located in any position throughout a protein; a subset of these minimotifs are C-terminal instances [4]. A query of this database identified 3,593 C-termini minimotif instances and consensus sequences for human proteins, representing a significant fraction of the human proteome.

In addition, 867 minimotif instances were inferred from rodent C-terminal minimotifs, which are generally highly conserved with human orthologs [43]. Consensus sequences (n = 47) for functional C-termini were obtained from MnM3. Information for integration with other external databases, including the PDB, UniProt, and PubMed is provided [9,35,36]. The relationships between the data sources are shown in an entity-relationship diagram (**S2 Fig**).

### Functional landscape of the C-terminome

The C-terminome database contains 3,593 functional minimotifs in the human proteome supported by experiments published in the literature. This is comprised of 47 consensus sequences and 3,546 minimotif instances. Based on recent estimates of ~21,000 protein coding genes in the human genome, known C-terminal minimotifs are found on at least 13% of the protein coding genes [44]. Stratification of the functions of all 3,593 minimotifs shows that the majority are involved in posttranslational modification (PTM), with fewer involved in binding interaction and a small percentage involved in protein trafficking (**Fig 1**). 23 different types of C-terminal PTMs were observed, the majority of instances being for phosphorylation (~71%) with a significant fraction of amidation, proteolysis, acetylation, and ubiquitination. Most PTMs had frequencies less than 2%. Most binding motifs were targets of proteins with PDZ domain (91%) and there were four other binding domains with less representation. Seven percent of binding motifs in the database had targets, but the binding domain was unknown. Trafficking motifs for seven organelles were represented. Several of the C-terminal minimotifs have more than one molecular function (n = 35; **S1 Table**).

### Predicted functional minimotifs in the C-terminome

Two proven approaches for predicting new C-termini functions were implemented: 1) inferring function from experiments on orthologous mouse and rat protein C-termini; and 2) predicting new instances from known consensus sequences.

**Fig 2:**
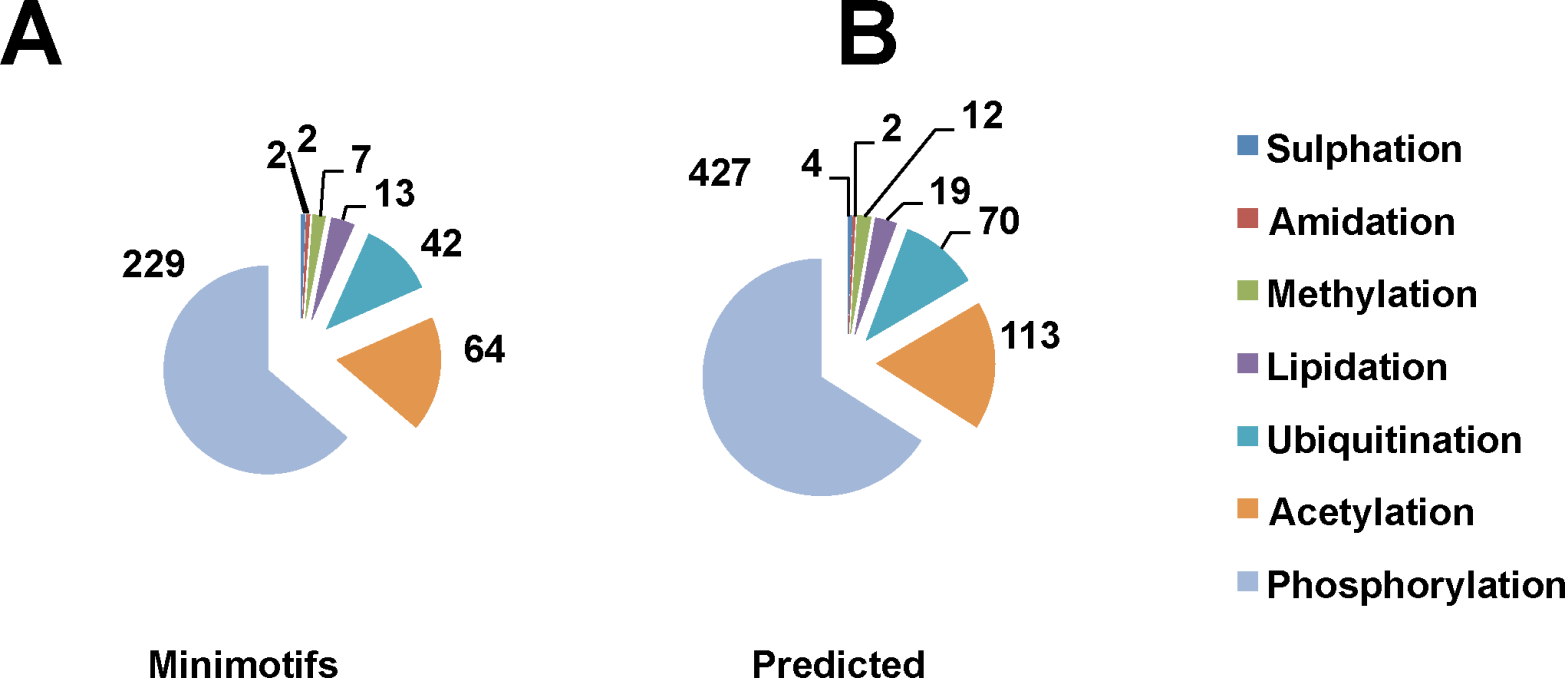
Predicted C-terminal PTM minimotifs inferred from rodent orthologs. Pie charts show the number and types of instances of (**A)** known minimotifs in rodent proteome and (**B**) predictions using consensus sequences derived from rodent cell experimentation (see keys).

Most of C-terminal minimotifs identified in rodents have highly conserved sequences in the human orthologs. Thus, as this strategy is also implemented by UniProt, it is fairly safe to infer function in the human protein [9]. There are 867 C-terminal minimotif instances in mouse and rat proteomes, which are conserved in a human orthologs, but do not yet have an annotation for a human protein (**Fig 2**). The majority of these instances were for seven types of PTMs and 220 were for binding to PDZ [43].

**Fig 3:**
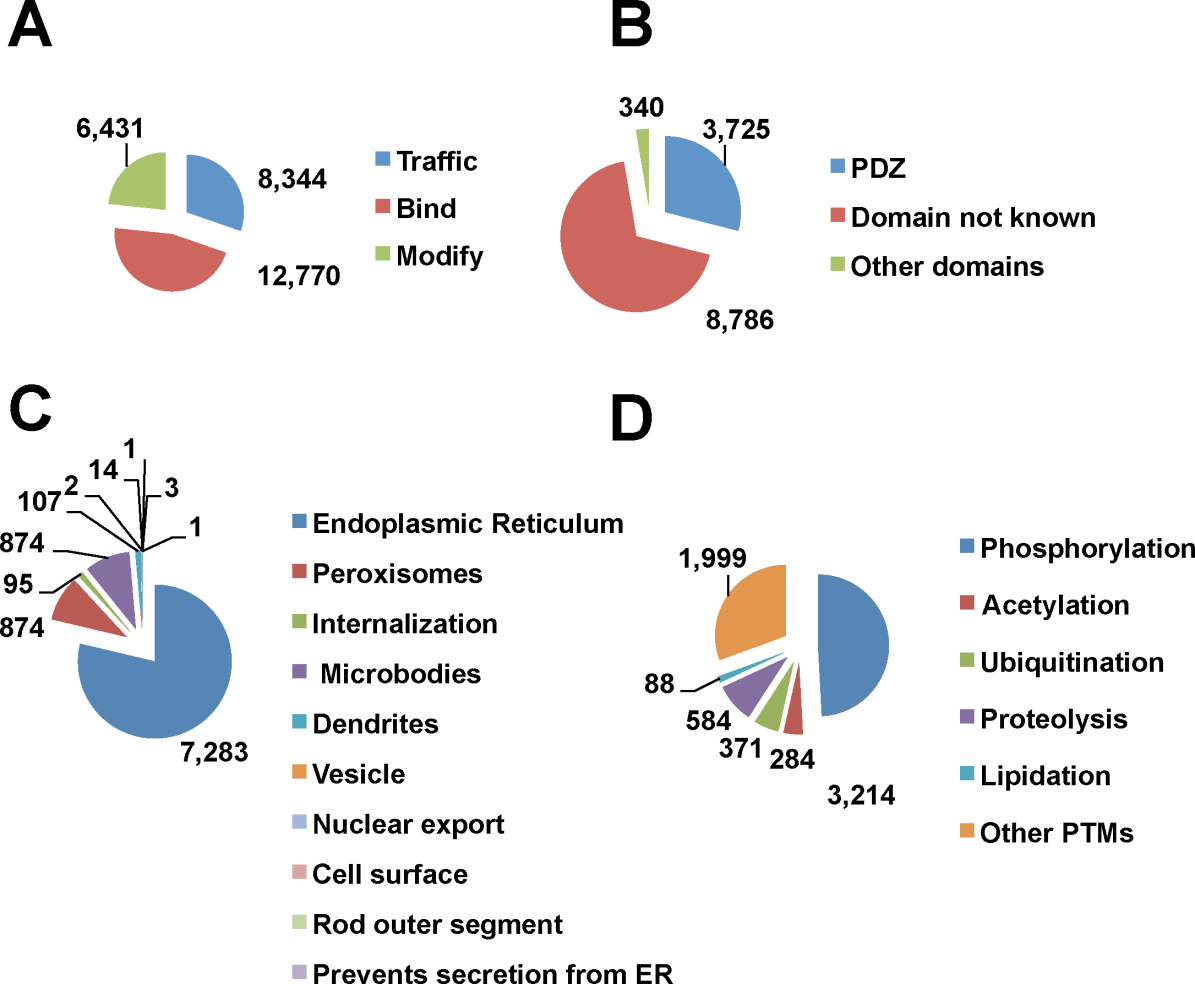
Predicted C-terminal instances from minimotif sequences. Pie charts showing predicted instances from matches to minimotif sequences for three major minimotif categories (**A**), binding minimotif types (**B**), PTM types (**C**), and trafficking motif types (**D**). The “Other domains” and the “Other PTMs” are as described for **Fig 1**. (**D)** The “Other” category includes trafficking to vesicles, nuclear export, cell surface, rod outer segment and prevents secretion from ER.

In addition to the direct inferences of instances from rodent data, consensus sequence matches are a source of new predicted minimotifs in humans. While these predictions are generally associated with a high false positive rate, anchoring a minimotif on the C-terminus increases the accuracy of predictions [45]. The 47 known consensus sequences and 3,546 instances from MnM3 database were used to predict new minimotifs on the C-termini. These 3,593 C-termini minimotifs were used to search the human proteome generating 27,546 new predictions (**Fig 3**). While most of the consensus sequences are for different types of PTMs, this functional class had the fewest predictions. A breakdown of the numbers of different functional categories for the predicted instances is shown in **Fig 3**. For the mouse and rat consensus sequences, another 220 human PDZ-binding minimotifs and 647 PTMs were inferred (**Figs 2** and **3**). The most common consensus sequences with the number of predictions is shown in **Table 2**.

**Table 2:** Verified functional C-termini consensus sequences.

Consensus Sequence	Molecular Function	Description	PubMed Identifier	# Predicted Minimotif Instances^3^	# Total Instances^4^
^1^x[D/E]x[AVILMFYW]> ^2^	Bind	PDZ domain class III binding	11741967	721	723
x[AVILMFYW]x[AVILMFYW]>^2^	Bind	PDZ domain class II binding	11741967	696	702
[KRHQSA][DENQ]EL>	Bind, Traffic	KDEL receptor binding motif	3545499	80	81
x[S/T]x[AVILMFYW]>^2^	Bind, Traffic	Peroxisomal targeting	1567655	108	111
[ST]x[LV]>	Bind	PDZ domain class I binding	11741967	1,432	1,441
[STAGCN][KRH][LIVMAFY]>	Bind, Traffic	Peroxisomal targeting	2901422	805	839
[WFY]RP[WFY]x(0,6)>	Bind, Traffic	Endoplasmic reticulum (ER) export	8649374, 12972562	114	116
C[AVLIFYWM][AVLIFYWM][ACDEFGHIKNPQRSTVWY]>	PTM	Farnesylation	8702508 2187294	104	109
C[AVLIFYWM][AVLIFYWM][LM]>	PTM	Geranyl-geranylation	8702508 2187294	70	71
Cxxx>	PTM	Farnesylation	1903399	178	180
C[GAVLI][GAVLI]x>	PTM	Prenylation	8702508	304	306
CxxM>	PTM	Mevalonation	2686979	47	48
DEWDx>	Bind	Aldolase binding	16278221	0	1
DxE>	Bind, Traffic	COPII binding	11726510	131	132
FFxxKKxx>	Bind, Traffic	Arf1 binding motif	15125774	2	3
FxxxFxxxF>	Bind, Traffic	ER export	11331877	2	3
Kx(0,1)Kx(1,3)>	Bind, Traffic	ER retention	2120038	1,544	1,548
S[ST]L>	Bind	PDZ domain class I binding	11741967	84	85
SxS>	Bind	Phosphorylation of Smad	9346966	404	405
VxPx>	Bind, Traffic	Rod outer segment trafficking	15728366	101	102
(V/L)xxSL>	Bind, Traffic	Cell surface expression of Kv1 family K^+^ channels	11343973	10	11
Yxx[AVILMFYW]>	Bind, Traffic	Lysosomal targeting, Dendritic targeting	9175836, 15689548	94	98
VMI>	Traffic	ERGIC compartment export	14517323	0	1
LxxLxPDExD>	Traffic	Glut4 targeting	24939910	0	1
FF>	Bind, Traffic	Endoplasmic Reticulum Export	9395526	78	79
HDEL>	Bind, Traffic	Internalization	2178921	12	14
KDEL>	Bind, Traffic	Nuclear export, To cell surface & dendrites	3545499	11	14
KKx>	Bind, Traffic	To Endoplasmic Reticulum Import	2120038	295	296
**TOTAL**				7,427	7,520

^1”^x” indicates any of the twenty amino acids and “> “designates the C-terminal end of a protein [1,46]

^2^Although a more specific consensus specificity profile for the PDZ domain recognition exists, a more simplified classification was used [28,43,47,48].

^3^Predicted minimotif instances are matches to consensus sequences that have not yet been experimentally tested.

^4^Total instances include both predicted and experimentally verified minimotif instances.

### Identification of new highly represented C-terminal sequences

**Fig 4:**
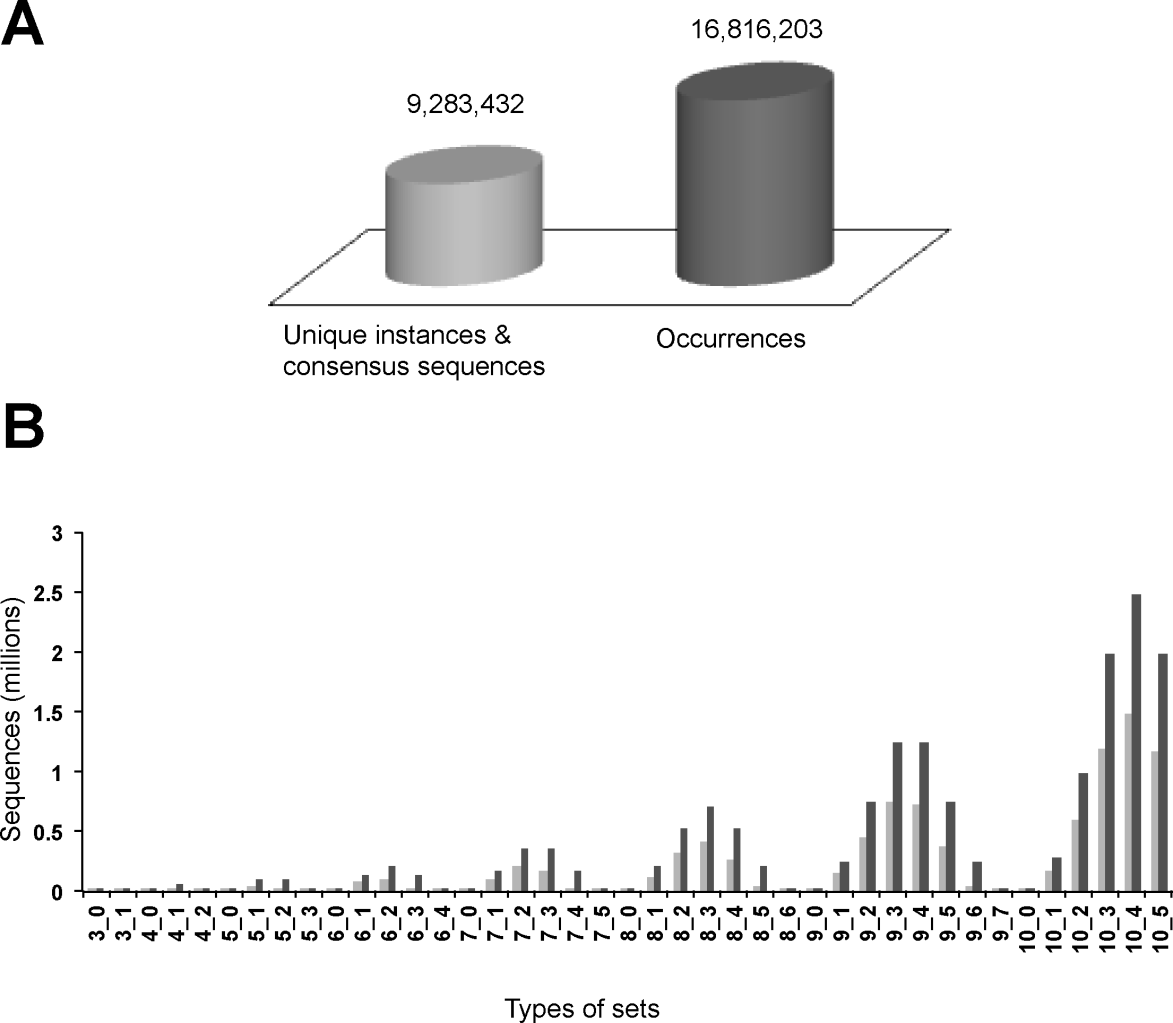
*de novo* consensus sequence matches in the human proteome. Consensus sequences and instances of length 3–10 residues and up to 5 degenerate positions were generated and used to search the last 10 amino acids of each protein in the human proteome (**A**). This included alternatively spliced isoforms. The inset shows the total number of sequences searched and number of occurrences identified. For each set of sequences with a given length and degeneracy (e.g. 3–1), the number of sequences searched and occurrences identified are shown in the bar graph.

Given that there are many C-terminal minimotifs in the human proteome, we thought there might be C-terminal minimotifs yet to be discovered. Previous efforts had searched for enriched sequences on the C-termini of proteins, but only looked for 3mers or 4mers or included limited degeneracy at only one position [20–23]. Here, the human proteome, including the splice variants, was analyzed for consensus sequences present in the last 10 amino acids of each protein **(Fig 4A and 4B)**. A total of 9,283,432 predicted consensus sequences and instances are 3–10 residues in length and with 0–5 completely degenerate positions. These were generated and used to search the proteome. Our set nomenclature lists the length and number of redundant sequence positions. For example, a set of “4–2” implies a four amino acids long consensus sequence with two degenerate positions. Matches to these consensus sequences and instances yielded a total of 16,816,203 occurrences in the human C-terminome. The number of sequences searched and occurrences identified are shown in **Fig 4A and 4B**. As expected, consensus sequences with more degenerate positions produced more matches.

### Ranking and selecting minimotif predictions

**Fig 5:**
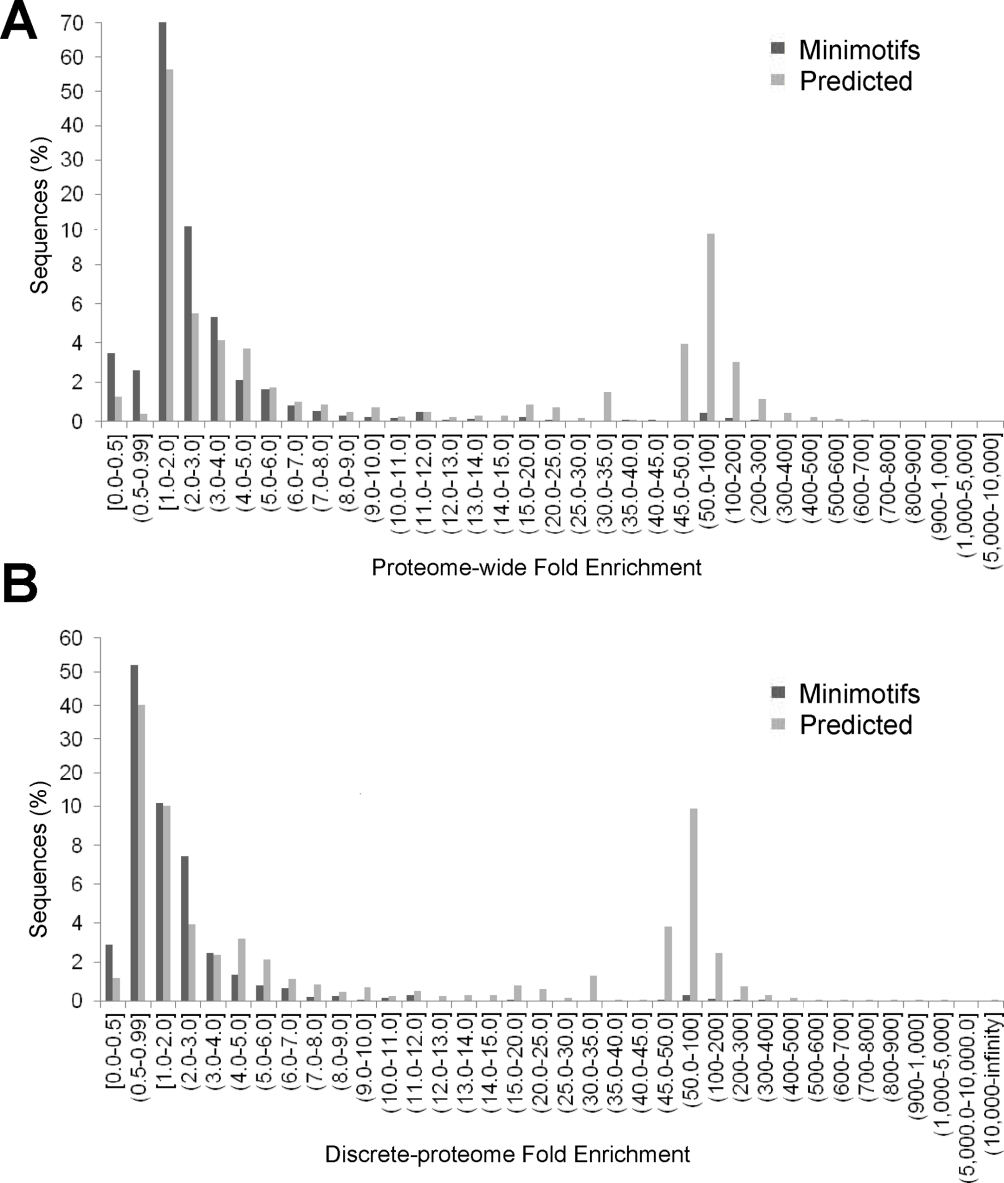
Fold-enrichment scores of minimotifs and predicted sequences. Bar graph showing the percentage of occurrences with different proteome-wide (**A**), and discrete-proteome (**B**) fold-enrichments. Dark gray bars represent the percentage of C-terminal minimotifs, and light gray bars represent the percentage of predicted consensus sequences and instances from *de novo* generated sequences.

We needed an approach to rank predictions. The Sig statistic was first used to assess minimotif predictions, but was too computationally intensive for the large number of predicted minimotifs [47]. Therefore, proteome-wide and discrete-proteome fold-enrichment scores were assessed using a set of C-termini minimotifs as true positive and experimental null mutants as true negatives (**Fig 5A** and **5B**). These scores are implemented on the web system. However, both discrete and proteome-wide scores of true negatives and true positives were not significantly different, likely because of the small number of minimotifs where a true negative could be identified [Mann-Whitney U test (n_TP_ = 75, n_TN_ = 21, p<0.05); for the discrete proteome (μ_TP_ = 9.0, μ_TN_ = 14.8, U = 559, p<0.05); and proteome wide (μ_TP_ = 37.2, μ_TN_ = 7.3, U = 1785, p<0.05)] (**S2 Table**).

Due to the limitations in statistics for evaluating the validity of new minimotif predictions, we needed some means to rank minimotif predictions. GERP scores are an accepted metric for sequence conservation and selection [39]. GERP scores were added for all proteins in the human proteome (10 amino acid C-terminus). GERP scores are also added to the results pages in a color-coded scheme and hovering the mouse reveals numeric scores. A color key is displayed on the website. The conservation metric can be used to identify which residues in a minimotif are more constrained (scores > 2), and thus more critical for function [48].

A total of 225 minimotif instances of SKL>, KDEL>, VPV>, and C[GAVLI][GAVLI]x> were analyzed to determine whether GERP scores are a good metric of minimotif prediction specificity (**S3 Table**). Specificity was assessed by PPV (Eq 1) and Accuracy (Eq 2). In order to calculate accuracy (Eq 2), TP and TN are needed; however, TN could only be identified for the SKL> and KDEL> minimotifs, thus only 99 minimotif instances were used. Both an average GERP score for each position in the minimotifs and a minimum GERP score for all position in each minimotif were evaluated.

**Fig 6:**
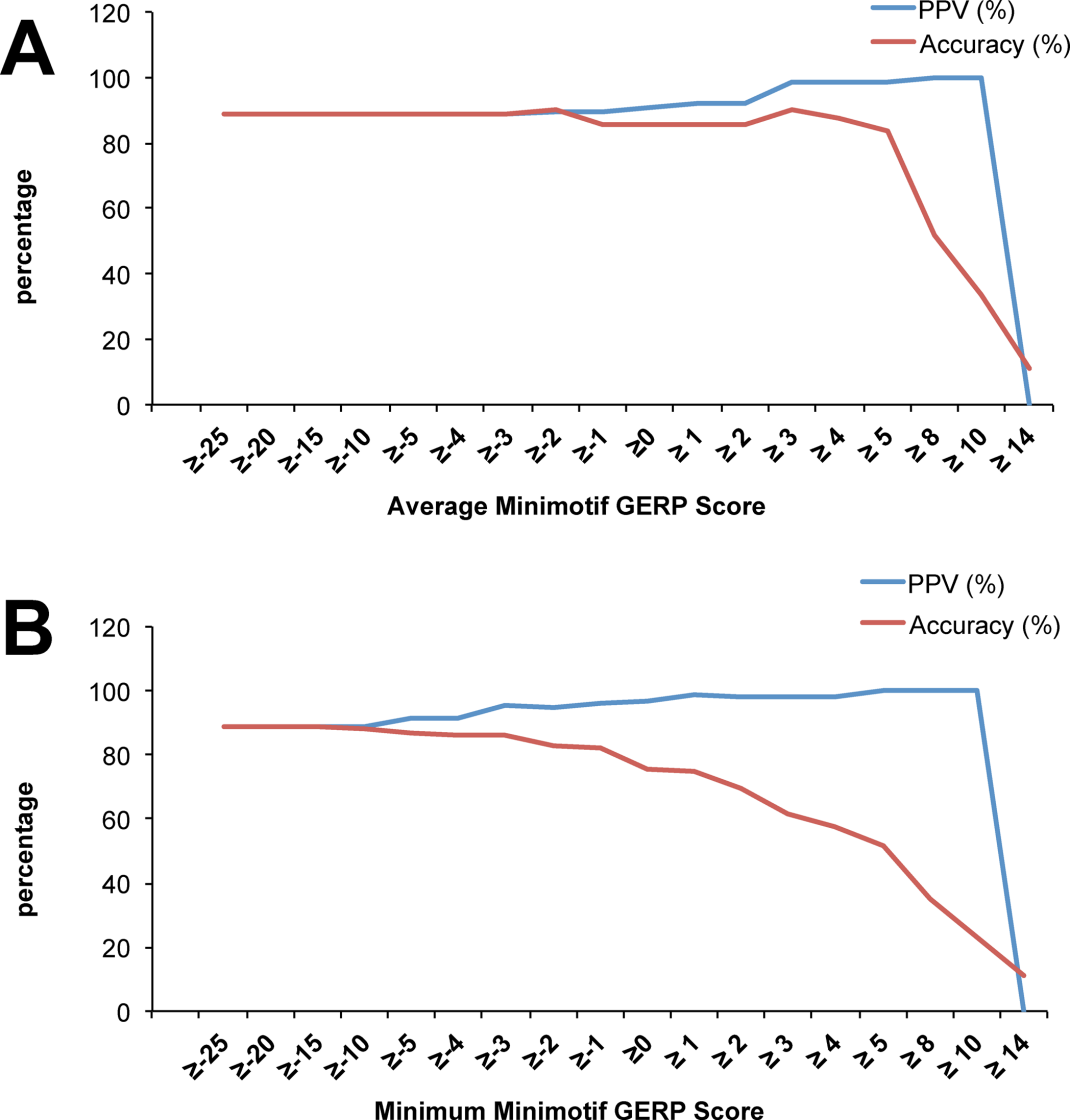
GERP score analysis of minimotif prediction. Line graph showing the percentage of positive predictive values and accuracy for average minimotif GERP score **(A)**, and minimum minimotif GERP score **(B)** thresholds.

Both the PPV and accuracy plateaued with an average minimotif GERP score of 5, which had good accuracy and PPV. However, we recommend use a threshold score of 2 on the web system because it also has good accuracy and PPV, is likely to have higher sensitivity, and is the published threshold previously used to analyze 1000 genomes data (**Fig 6**)[48]. The average GERP score threshold of 2 produced 92% PPV with an accuracy of ~86% (**S4 Table**). Similar results were obtained with the minimum minimotif GERP scores was used. We wanted to measure sensitivity, but could not because we do not have any false negatives, thus relied on the specificity evaluation. Thus, it appears that GERP scores are good metrics for minimotif prediction specificity. The user can select any threshold on the C-terminome web system.

### C-terminome web application

**Fig 7:**
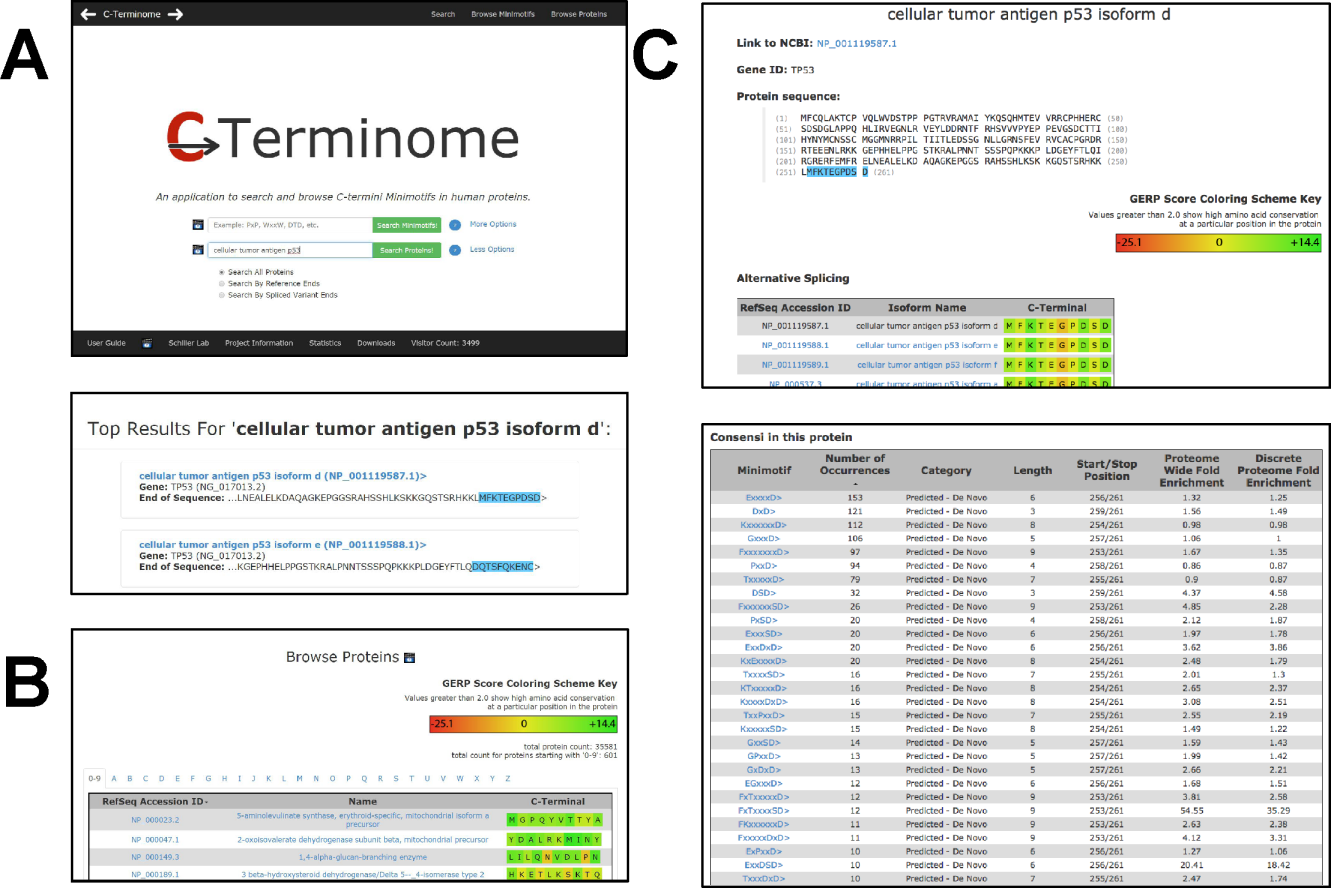
C-terminome protein search and browse pages. **(A)** The main C-terminome search page can be used to select, search and browse proteins or minimotifs. Question marks open a popup window with acceptable syntax. When a protein is entered into the textbox and a search is initiated, a results page shows the top hits for the protein search term (bottom panel). Presented information includes the RefSeq protein accession number, gene name, and protein sequence with the highlighted C-terminus. **(B)** A protein can also be selected from the Browse Proteins page. This list can be searched for protein names alphabetically or browsed for C-termini of different proteins. A key at the top indicates the GERP score for each residue at the C-termini. **(C)** Both Search and Browse Proteins produce a page with the results shown in **C**. This includes the RefSeq protein accession number, protein name, protein sequence with the C-terminus highlighted, and alternative spliced variants with the RefSeq accession number, and isoform name (top panel); a list of consensi present in the protein including whether the consensus sequence was experimental or predicted, number of instances, and both proteome-wide and discrete proteome fold enrichment (bottom panel).

The C-terminome web system can be used to retrieve information about C-terminal minimotifs using three functions accessible from links in the title bar of the main search page (**Fig 7A**): 1) Search minimotifs or proteins (default); 2) Browse Minimotifs; and 3) Browse Proteins. The link-bar on the bottom of this page redirects the user to project information, video tutorials, and a user guide with examples. In addition, users can email their comments and suggestions to improve C-terminome. The C-terminome minimotif data is available as a SQL dump download at http://cterminome.bio-toolkit.com and from FigShare at https://dx.doi.org/10.6084/m9.figshare.3082027.v1.

#### Search and browse proteins pages

The search page (default load) can be used to search for C-terminal minimotifs by minimotif sequence or protein (**Fig 7A**, top panel). The main area of the search page contains two search text boxes, one for searching by minimotif sequence and another for searching by protein name or RefSeq accession number. “More Options” hyperlinks reveal radio buttons to enable search selections for proteins with reference ends or alternative spliced ends. Several options are available search for minimotifs, or predictions by several approaches. Selection of the question mark next to these textboxes describes the acceptable syntax. Entry of incorrect syntax displays an error message.

The Browse Proteins page displays all protein and their isoforms with RefSeq accession number, protein name, and the C-termini (last ten residues) of a protein (**Fig 7B**). Proteins are alphabetically organized, but the menu bar can be used to sort entries facilitating easy navigation. A key to the GERP scores indicating the conservation of each residue in the C-terminal region of proteins is given at the top. A pop-up over the amino acid reveals the GERP score. Each entry on this page is linked to a results page with general information, alternative spliced variants, and C-terminal consensus sequences (**Fig 7C**). This information includes the RefSeq number, gene symbol, and sequence with C-terminus highlighted (**Fig 7C**, top panel). The results page also contains alternative spliced variants for the selected protein, which can alter the minimotif(s) present on the C-terminus. Shown also is the C-termini of each splice variant, as exemplified for a number of different TP53 isoforms. All C-terminal consensi sequences present in the selected protein are shown (**Fig 7C,** bottom panel), including those that are minimotifs or predicted minimotifs, and each C-terminal motif is then linked to a results page with more detail.

#### Browse Minimotifs page

**Fig 8:**
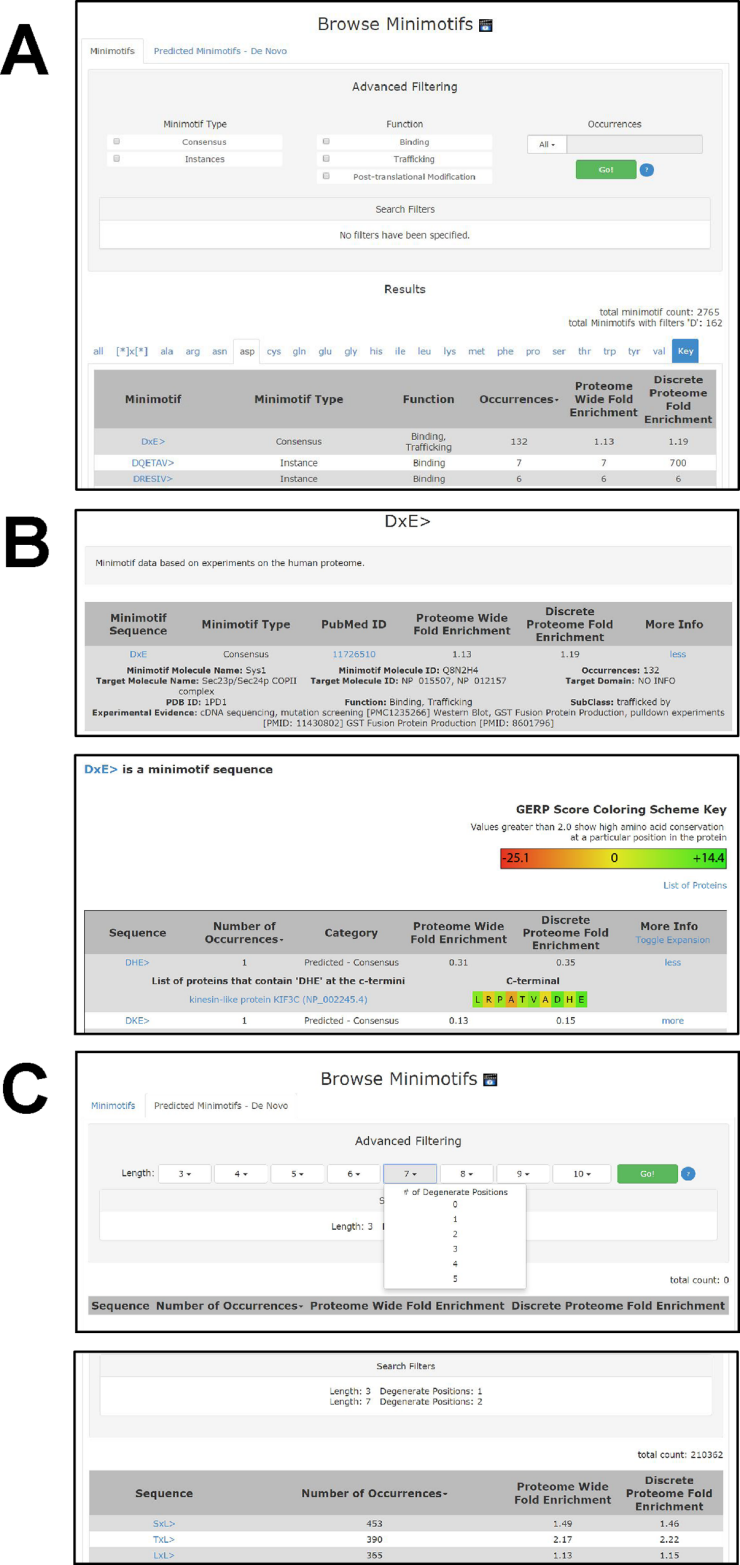
Browse C-termini minimotifs and predicted minimotifs page. The Browse Minimotifs page has two tabs for searching different types of data: C-termini minimotifs, and predicted minimotifs. **(A)** The main display for the minimotif instances and consensus sequences tab has several different motif type and function selectors for identifying minimotif instances. **(B)** An example of a minimotif result page shows the information provided for an identified minimotif (top panel). If you mouse-click the motif sequence hyperlink, it reveals the set of minimotifs with that consensus sequence (bottom panel). **(C)** Browse Predicted Minimotifs page for *de novo* sequences that are highly repetitive in the human proteome. Top panel shows the main selector display page, where the length and degeneracy of the sequence is chosen. A list of minimotifs produced from the search is shown in the bottom panel. Sequences are hyperlinked to more information about the predicted minimotif.

On the Browse Minimotifs page, two tabs with C-termini minimotifs (default load) or those predicted by matching a novel consensus minimotif (**Fig 8A**).

#### C-termini minimotifs tab

This tab has a sortable minimotif list and set of selection filters for functional C-terminal minimotifs identified by experimentation (**Fig 8A**). The list of minimotifs with motif type, function, and occurrences can be sorted using the column name in the title bar. The default sort is based on the first amino acid of the sequence (**Fig 8A**). Each hyperlinked minimotif sequence provides information about its molecular function, instances of the motif in the proteome and other attributes (**Fig 8B**). The “more” link expands each minimotif section (**Fig 8B,** top panel).

#### Predicted-*de novo* minimotifs tab

The predicted minimotif page is organized like the C-termini minimotifs page, but its minimotifs were algorithmically predicted (**Fig 8C**). The filters for predicted minimotifs includes the length of the sequence and number of degenerate positions in the sequence **(Fig 8C**). The results for selection 5–1 and 7–2 set are shown in **Fig 8C** (bottom panel). The minimotif sequences are linked to display information similar to that in **Fig 8B** (bottom panel).

#### Minimotif search

**Fig 9:**
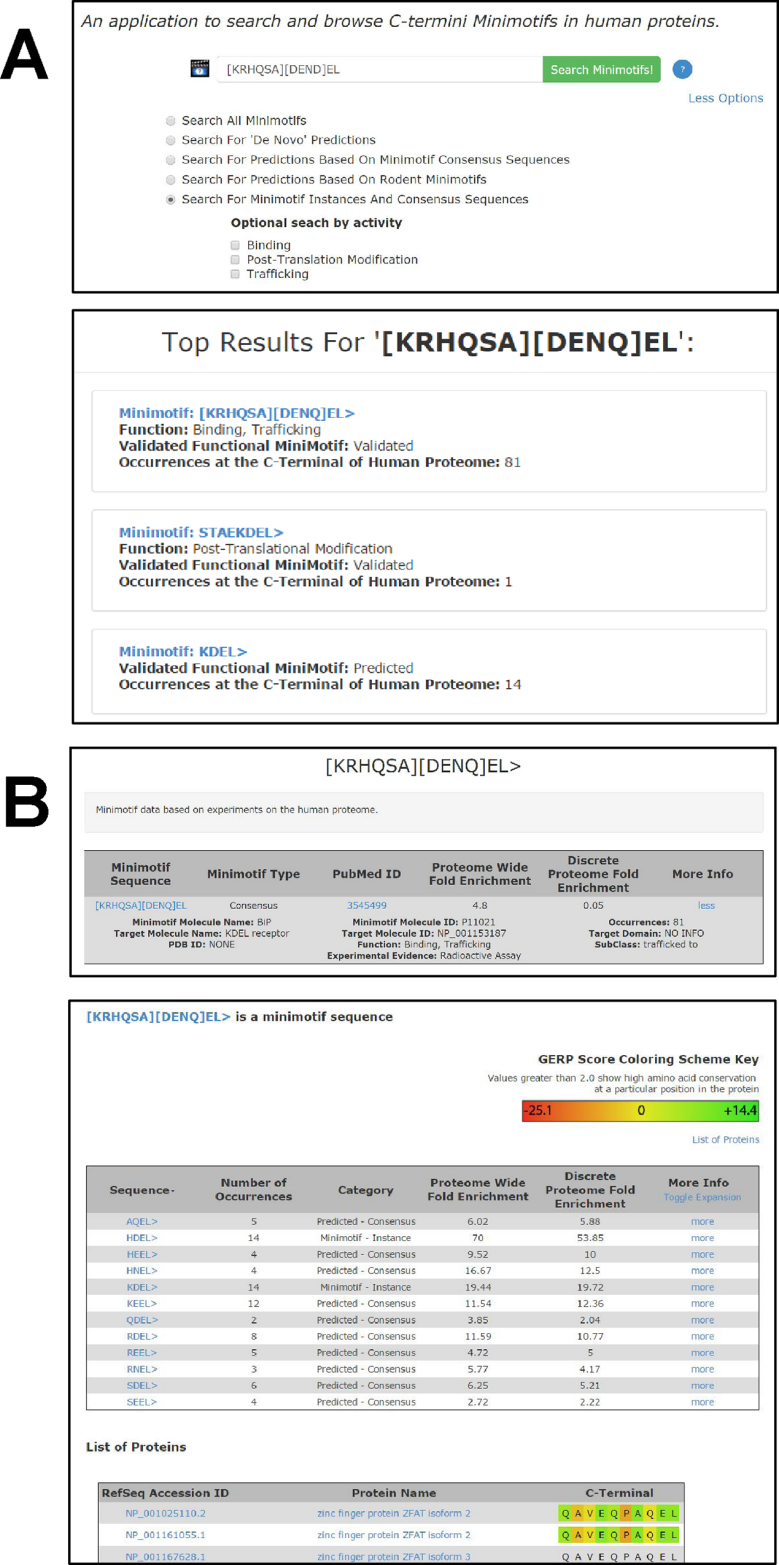
Search minimotifs page. **(A)** When a minimotif is entered into the search page textbox (top panel) and a search is initiated, it produces a results page with the top hits for the consensus sequence (bottom panel). Presented information includes information about how the motif was identified and how many times it occurs in the human proteome. The search can be restricted to prediction or minimotif instances. Selecting the question mark open a popup window with acceptable syntax. **(B)** Once a particular minimotif is selected, a new results page displays more specific information. Selecting one of the sets of instances reveals a list of proteins containing the consensus sequence and the C-termini of these proteins.

In addition to being browsed, minimotifs can be searched by a consensus sequence from the main search page (**Fig 9A**, top panel). Minimotif types (e.g. minimotif instances, *de novo* predictions, predictions based on rodent proteome, or consensus sequence predictions) can be selected with radio buttons in the “More Options” hyperlink. The search produces a list of motifs that contain the sequence entered (**Fig 9A**, bottom panel). Selection of a sequence hyperlinks to a new page with more information (**Fig 9B**).

#### User guide and video tutorials

The web application has a home page with links to a user guide. The guide contains instructions, the data model, calculations, and example analyses and workflows. Video tutorials are provided to help understand the capabilities of the web system.

### Variability and selection of minimotifs in the human population

**Fig 10:**
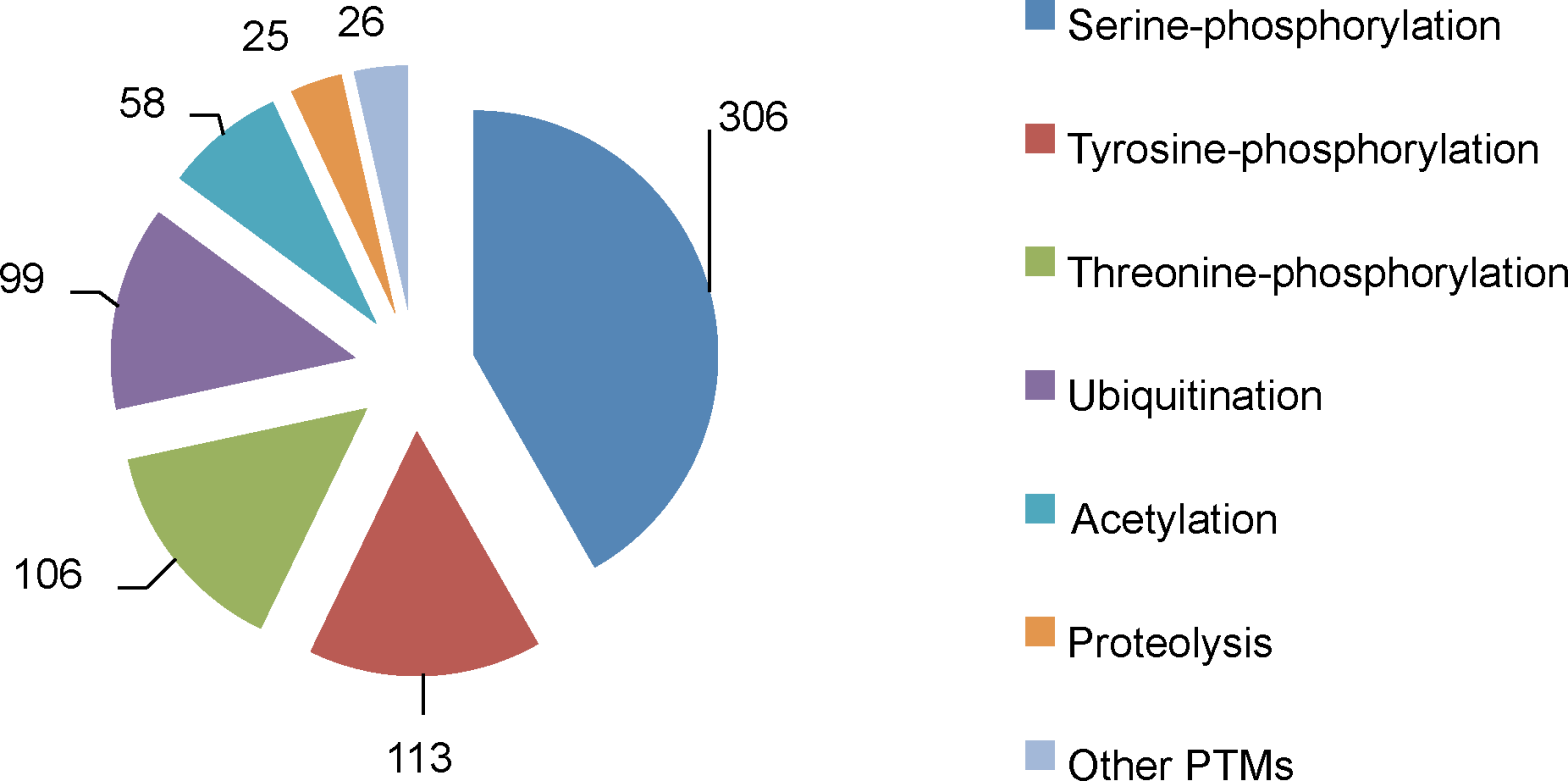
Variability of C-terminal minimotifs in the human population. The pie chart represents breakdown of functional classes of 730 single nucleotide polymorphisms (SNPs) identified in C-terminal minimotifs post-translational modification. The breakdown of the modification function is shown in an extended pie chart. The “other PTMs” category includes methylation, prenylation, crotonylation, glycosylation, lipidation, and sumoylation minimotifs. PDZ domain binding motifs (1%) are not shown.

Since C-terminal minimotifs are key functional elements in proteins, we examined how variable they were in the human population using data from the 1000 genomes project (phase I) [48]. 736 single nucleotide polymorphisms (SNPs) were identified in 650 minimotifs, indicating that 82% of the experimentally verified C-terminal minimotifs are largely fixed in the human population, while a smaller subset is variable. 99% of the variants were in different types of C-terminal PTM minimotifs (**Fig 10**) and 1% were in the PDZ domain binding minimotifs.

Approximately half of the variants (333) encoded non-synonymous substitutions. Since some variants were in key consensus positions where an amino acid is covalently modified, these are assigned as loss of function variants. For example, if a key lysine in a ubiquitination site is mutated, ubiquitin cannot be covalently attached to the protein at this position. Twenty loss-of-function variants in C-terminal minimotifs were identified, mostly in serine- and threonine- phosphorylation sites, and few proteolysis and ubiquitination sites (**S5 Table**).

**Fig 11:**
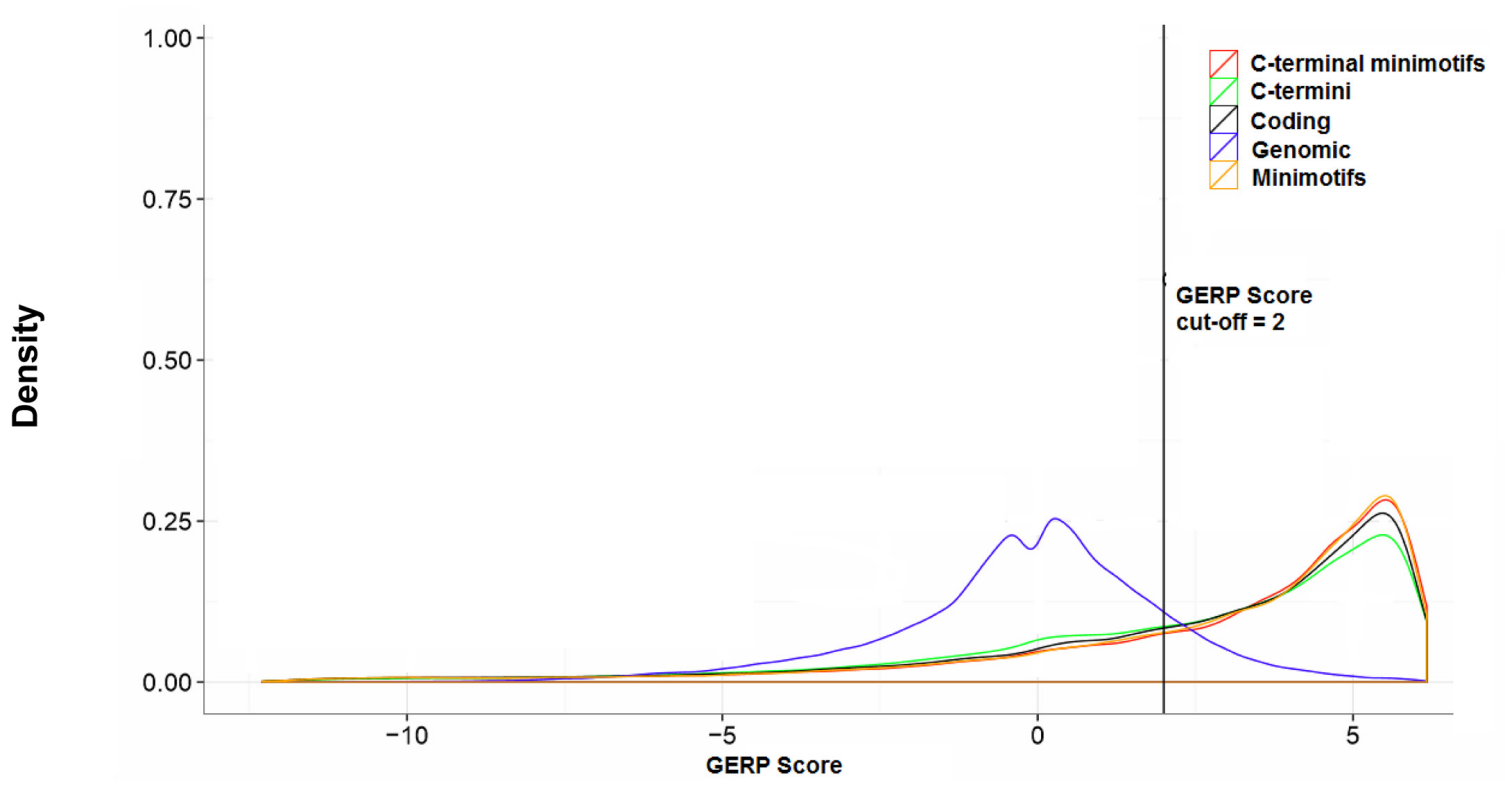
Selection of C-terminal minimotifs in humans. Density distribution plots showing the GERP scores for different genomic regions.

Selection of variants for C-terminal minimotifs was assessed (**Fig 11**). The genomic evolutionary rate profiling (GERP) score was used as a metric of minimotifs to identify minimotifs under negative selection as previously described [39]. Most C-terminal minimotifs had GERP scores above 2.

## Discussion

In this paper, data for the human functional C-terminome was collected and analyzed. To our knowledge, a generalizable function for the C-terminus of proteins has not been explored at the proteome level. Consolidation of C-terminal minimotif data revealed 3,593 previously known C-terminal minimotif instances. 13% of all genes encode a functional C-terminus indicating that a significant portion of genes express proteins with a functional C-terminus.

While the C-terminome database and web system is the first consolidation of C-terminal minimotifs functions and consensus sequences in human proteome, this effort is synergistic with ProTeus and TopFIND, two other databases that include the C-terminus as part of their focus [11,21]. Several additional concepts and functionalities make this system unique. The most important distinction is that the C-terminome web system focuses on anchored consensus sequences and instances. This is well justified because for most different types of C-terminal minimotifs, internal minimotifs are generally not observed suggesting that spacing to the charged C-termini is critical to their function.

The C-terminome database includes 650 binding, 44 trafficking, and 2,937 PTM minimotifs in the human proteome (**Fig 1**). ProTeus covers only predicted consensus sequences (or signature sequences) anywhere in the last 10 amino acids of the proteins and does not cover function. TopFIND, on the other hand, identifies only a small set of post-translational modifications on the C-termini (37 annotations currently) that are not specific to the C-termini. Other minimotif functions such as binding and trafficking are not included in TopFIND. Furthermore, the C-terminome database also covers alternative splicing variants, which are relevant because alternative splicing introduces new C-termini and can alter the minimotifs in protein isoforms [7]. The new C-termini can also be derived from proteolysis. However, the C-terminome database does not yet cover these new C-termini as covered in TopFIND [11].

To our knowledge other web systems have not used this approach to extensively explore functional C-terminal minimotifs. Approximately 17% of the proteins in the proteome had a known function. C-terminal minimotifs inferred based upon minimotif consensus-based sequence predictions represented 30% of proteins in the proteome. Since rodent and human orthologs are highly conserved, we think it is fairly safe to use C-terminal minimotifs discovered in rodents to infer functions in human orthologs. 867 C-termini functions were inferred based on experiments done on the rodent proteome, but these predictions are a negligible percent of proteins. Although the new repetitive consensus sequence we identified cover the majority of the proteins in the C-terminome, these predictions are not associated with a function. Nevertheless the C-terminome website has minimotif instance or functional predictions that cover approximately half of the protein in the human proteome.

The identification of many additional repetitive consensus sequences on the C-termini suggests that there are likely many more to be discovered. Several groups have tried to identify repetitive consensus sequences on the C-termini of proteins. However, only ProTeus has explored C-terminus for consensus sequences using one degenerate position. We used more degenerate positions and anchored sequences. The known C-terminal minimotif consensus sequences presented in **Table 2** have 1–5 degenerate positions, which is often observed in the minimotifs in the Minimotif Miner database [4]. Thus, of the previous studies looking for consensus sequences on the C-termini with zero or one degenerate positions, few are likely to be highly selective for functional minimotifs [20–23]. This is why an algorithm was designed to search for anchored minimotifs of length 10 with 0–5 degenerate positions. It is important to stress that identifying consensus sequences only infers functions, and the functional relevance of these sequences will need to be tested.

Identification of C-terminal minimotifs using the C-terminome webs system is useful for several reasons. Identification of new minimotifs can help connect proteins having unknown or poorly understood functions with other proteins having well-defined roles in established pathways or cell processes. New C-terminal minimotifs introduced by alternative splicing can be identified. Proteins may share common modular minimotifs despite poor overall sequence identity because they have common binding partners, trafficking determinants, or PTM enzymes. For example, calcium-independent phospholipase A2-γ and acyl-coenzyme A thioesterase 8 have different molecular functions and only 10% sequence identity. However, they both contain peroxisomal-targeting SKL> motif and are located in peroxisomes [49–51]. Minimotifs discovery is also important because some are mutated in some human diseases and some may serve as targets of therapeutic intervention; there are several cases where minimotif mimetics are FDA-approved drugs [52–54].

## Supporting Information

S1 Fig Comparison of amino acids frequencies on C-termini to that in the proteome. The bar graph displays the frequencies of amino acids at the C-terminal region (the last 10 amino acids) and the entire human proteome (n = 35,581).
(TIF)

S2 Fig Entity-relationship diagram of C-terminome database. The ER diagram displays MySQL database tables with data fields and their associations. Each data source is a major table in C-terminome database and is associated through a primary key.
(PDF)

S1 Methods Method for generating *de novo* C-terminal sequence patterns. (DOCX)

S1 Table List of C-termini minimotifs having multiple functions. (XLS)

S2 Table List of mutants used in the experiments. (XLSX)

S3 Table Evaluation of the accuracy of GERP scores for predicting true positive minimotifs. (XLSX)

S4 Table GERP score analysis for minimotif conservation. (XLSX)

S5 Table Loss of function C-terminal minimotifs. (XLSX)
