# Supplementary figures and images for: The Functional Human C-Terminome
Source: PLoS One. 2016 Apr 6;11(4):e0152731. doi: 10.1371/journal.pone.0152731 (PMC4822787; doi:10.1371/journal.pone.0152731)

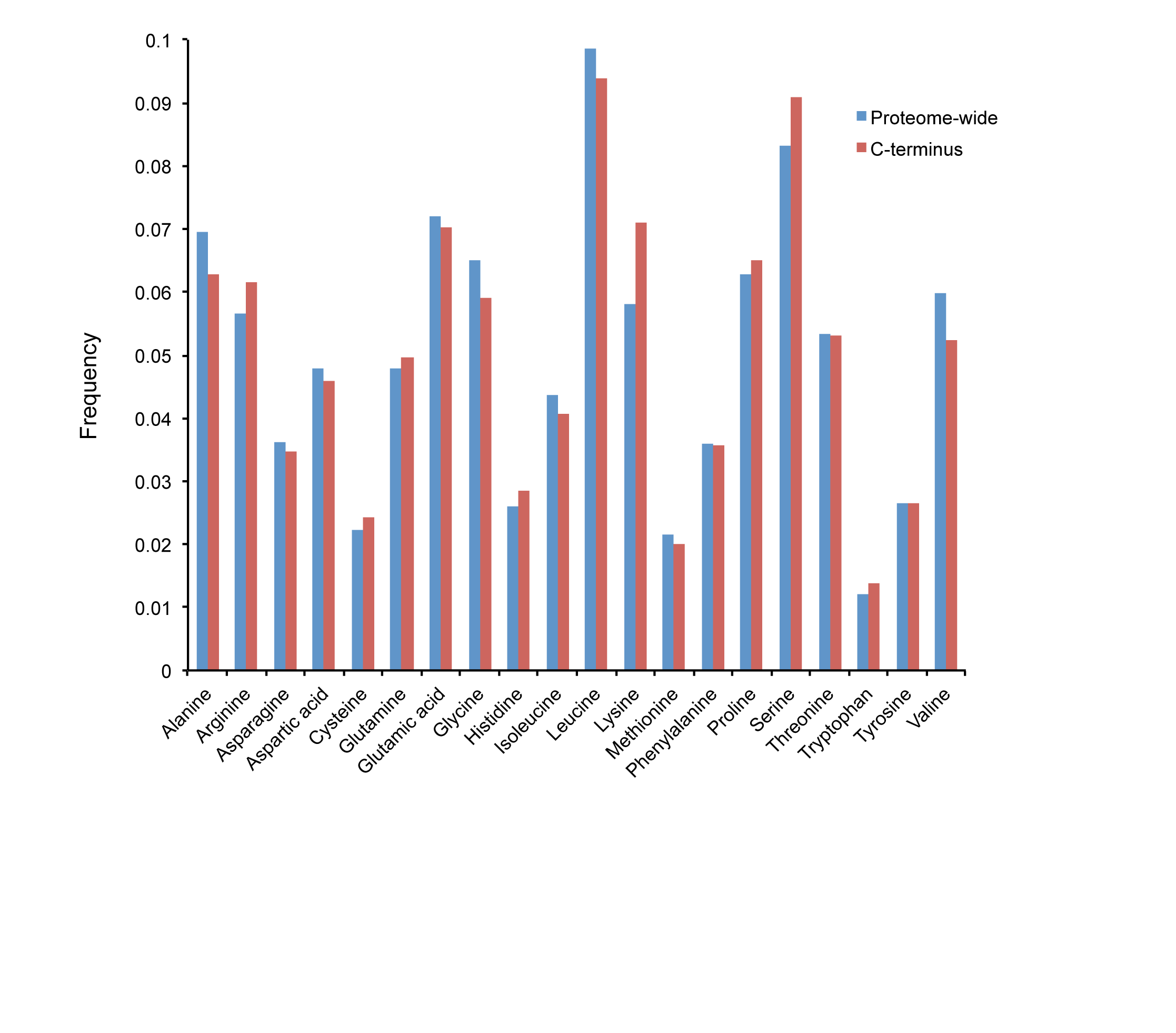

Supplement: S1 Fig — The bar graph displays the frequencies of amino acids at the C-terminal region (the last 10 amino acids) and the entire human proteome (n = 35,581). (TIF) [file pone.0152731.s001.tif]

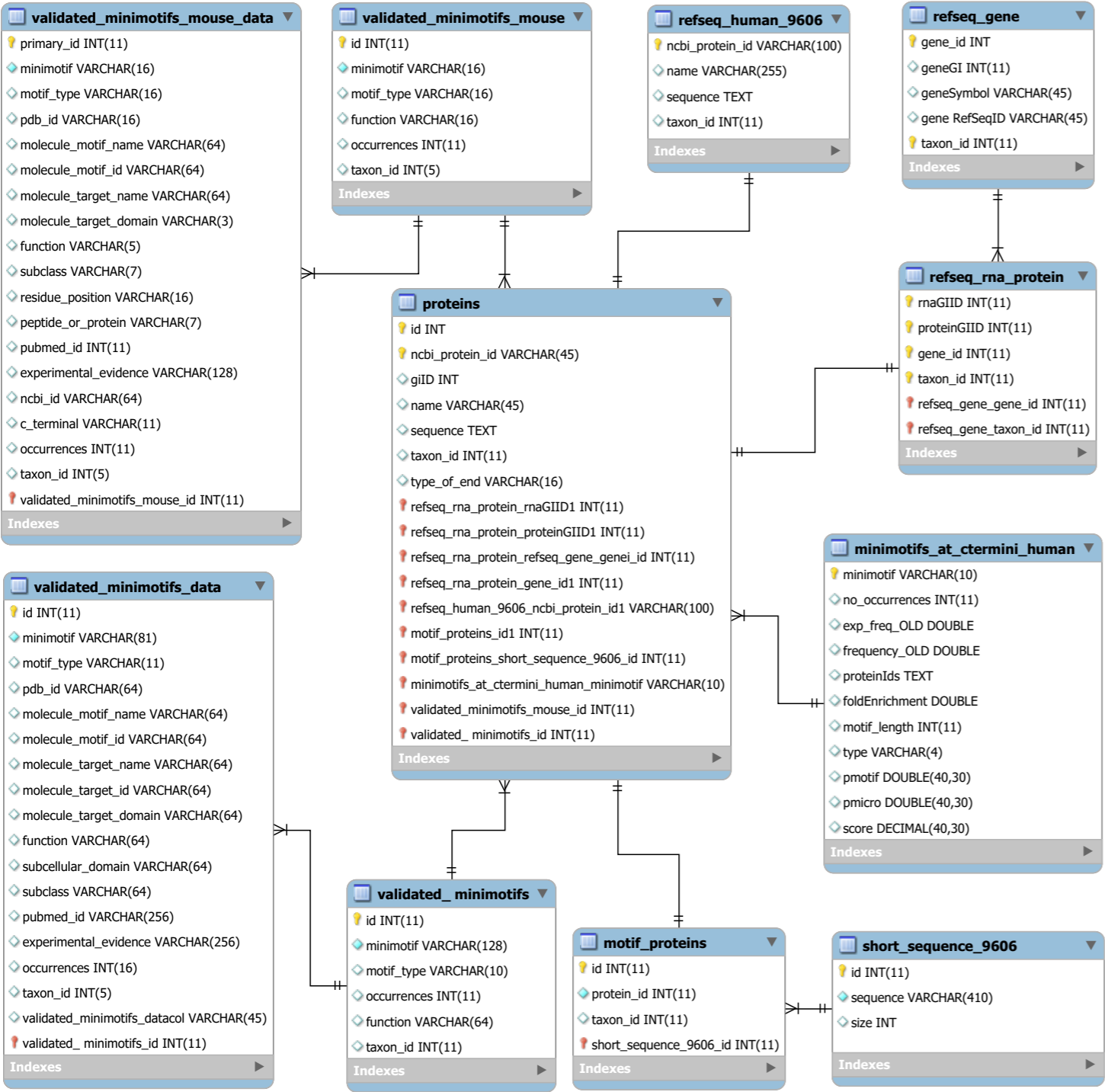

Supplement: S2 Fig — The ER diagram displays MySQL database tables with data fields and their associations. Each data source is a major table in C-terminome database and is associated through a primary key. (PDF) [file pone.0152731.s002.pdf]
