# Supplementary material for: The Functional Human C-Terminome
Source: PLoS One. 2016 Apr 6;11(4):e0152731. doi: 10.1371/journal.pone.0152731 (PMC4822787; doi:10.1371/journal.pone.0152731)
Supplement: S1 Methods — (DOCX) [file pone.0152731.s003.docx]

**S1 Methods. Method for generating *de novo* C-terminal sequence patterns.**

***Steps for generating sequences/patterns***

1. Get a list of protein sequences anchored at the c-termini between 3 and 10 amino acids in length
2. Generate terminal anchored combination supersets of the sequences generated in step 1
3. Eliminate supersets which do not occur more than once

***Step 1: Get a list of protein sequences anchored at the C-termini between 3 and 10 amino acids in length***

Let **Σ** represent the set of amino acids present in proteins of the human genome, then **Σ** can be expressed as:

**Σ = { A, R, N, D, C, E, Q, G, H, I, L, K, M, F, P, S, T, W, Y, V }**

A single element of the set **Σ** can be expressed as **σ_p_** where **_p_** represents the amino acid position in **Σ**:

**{ σ_p_ ϵ Σ, 1 ≤ p ≤ 20)**

**example: σ_19_ = Y**

A single element or no element can be symbolized as follows:

**{ σ_pε_ | σ_pε_ ϵ (Σ U empty set), 0 ≤ p ≤ 20, 0 = empty state}**

**examples: σ_3ε_= N, σ_0ε_ = { }**

Any generated sequence of length 3 to 10 amino acids can be represented as follows:

**ω = σ_a_σ_b_σ_c_σ_dε_σ_eε_σ_fε_σ_gε_σ_hε_σ_iε_σ_jε_**

**example: σ_12_σ_4_σ_6_σ_11_σ_0ε_σ_0ε_σ_0ε_σ_0ε_σ_0ε_σ_0ε_ = KDEL**

Let **Ρ** represent the set of proteins in the human genome, then **Ρ_i_** represents a single protein where:

**{ Ρ_i_ ϵ Ρ | 1 ≤ Ρ_i_ ≤ Ρ_SIZE_ }**

A position specific amino acid in protein **Ρ_i_** can be express as follows:

**Ρ_i, j_ = { σ_p_ | 1 ≤ j ≤ Ρ_i, LENGTH_ }**

**example: if Ρ_1_ = {…DMEETDD} then Ρ_1, LENGTH-2_ = T = σ_17_**

Let **Ψ** represent the set of C-termini sequences generated from **Ρ**, then **Ψ_i_**  is of the form **ω** where:

**Ψ_SIZE_ = (Ρ_SIZE_ * 8)**

**{ Ψ_i_ ϵ Ψ** **| 1 ≤ Ψ_i_ ≤ Ψ_SIZE_ }**

Generation of the set **Ψ**:

**for each Ρ_i_ ϵ Ρ**

**j = Ρ_i, LENGTH_ – 9**

**while( j ≤ (Ρ_i, LENGTH_ – 2) ) do the following:**

**ω = Ρ_i, j_ + _..._ + Ρ_i, LENGTH-1_ + Ρ_i, LENGTH_**

**add ω to Ψ**

**j = j + 1**

**example:**

**Ρ_i_** = {…GNMGPQYVTTYA}

**Ψ =** {MGPQYVTTYA, GPQYVTTYA, PQYVTTYA, QYVTTYA, YVTTYA, VTTYA, TTYA, TYA}

***Step 2: Generate terminal anchored combination supersets of the sequences generated in step 1***

A single degenerate position can be denoted by **Χ**, which represents all amino acids:

**Χ = { ∀σ_p_ ϵ Σ }**

A single degenerate position or no element at that position can be denoted as **Χ_ε_**:

**Χ_ε_ = { ∀σ_p_ ϵ (Σ U empty set) }**

A sequence with at least one degenerate position whereby neither the first nor the last position can be degenerate can be expressed as follows:

**φ = { σ_a_(σ_xε_ Χ_ε_)^*Χ (σ_xε_ Χ_ε_)^*σ_b_ | ^* symbolizes the shorthand expansion of (σ_xε_ Χ_ε_) }**

Let **Φ** represent the set of degenerate sequences derived from **Ψ**, then **Φ_i_** is a degenerate sequence of the form φ where:

**Φ_SIZE_ =** $\sum_{\boldsymbol{i}\boldsymbol{=}\boldsymbol{1}}^{\boldsymbol{\Psi}\mathbf{SIZE}} \sum_{\boldsymbol{r}\boldsymbol{=}\boldsymbol{1}}^{\boldsymbol{\Psi i}\mathbf{,}\mathbf{LENGTH}\mathbf{-}\mathbf{2}} \frac{\left( \boldsymbol{\Psi i}\mathbf{,}\mathbf{LENGTH}\mathbf{-}\mathbf{2} \right)\mathbf{!}}{\left( \boldsymbol{\Psi i}\mathbf{,}\mathbf{LENGTH}\mathbf{-}\mathbf{2}\mathbf{-}\mathbf{r} \right)\mathbf{!}\mathbf{*r}\mathbf{!}}$

**{ Φ_i_ ϵ Φ** **| 1 ≤ Φ_i_ ≤ Φ_size_ }**

Generation of the set **Φ**:

For each **Ψ_i_**

j=1

while ( j < 2**^Ψi, LENGTH-2^**) do:

φ **= Ψ_i, 1_**

mod_bin = j

k=2

while (k < **Ψ_i, LENGTH_**) do:

if the remainder of mod_bin/2 is 1, do:

φ **=** φ **+ Χ**

otherwise:

φ **=** φ **+ Ψ_i, k_**

mod_bin = mod_bin/2

k = k + 1

φ **=** φ **+ Ψ_i, LENGTH_**

add φ **to Φ**

j = j + 1

***algorithm b.***

**example:**

**Ψ** = { MGPQYVTTYA, GPQYVTTYA, PQYVTTYA, QYVTTYA, YVTTYA, VTTYA, TTYA, TYA }

**Φ =** { MXPQYVTTYA , MGXQYVTTYA , MXXQYVTTYA , MGPXYVTTYA, …, TXYA, TTXA, TXXA, TXA}

***Step 3: Eliminate supersets which do not occur more than once***

Let **Ω** represent a set of all degenerate and non-degenerate sequences derived from **Ψ** and **Φ**, then **Ω_i_** is either of the form **ω** or **φ** where:

**{ Ω_i_ ϵ Ω | 1 ≤ Ω_i_ ≤ Ω _SIZE_ }**

Generation of the set **Ω**:

for each **Ψ_j_**:

add **Ψ_j_** to **Ω**

for each **Φ_j_**:

count = 0

for each **Ψ_k_:**

if **Ψ_k_**  matches **Φ_j_** -> (note: all **Χ** in **Φ_j_** can be substituted for any amino acid at the same position in **Ψ_k_**)

count = count + 1

if count > 1

add **Φ_j_** to **Ω**

***algorithm c.***

**example:**

**Ψ = {** …, TGYA, ATYA, TTTA, TTRA, … **}**

**Φ = {** …, TXYA, TTXA, TXXA, …}

**Ω = {** …, TGYA, ATYA, TTTA, TTRA, …, TTXA, TXXA, … }
